# Acute Psychosis and Suicidal Attempt in Hashimoto’s Thyroiditis: A Case Report

**DOI:** 10.7759/cureus.71421

**Published:** 2024-10-14

**Authors:** Syed Ali Bokhari, Ghina Alramahi, Mostafa Abulmagd, Noura Alabrach

**Affiliations:** 1 Psychiatry, Al Amal Psychiatric Hospital, Emirates Health Services, Dubai, ARE

**Keywords:** antipsychotic medication, autoimmune thyroiditis, hashimoto’s thyroiditis, hypothyroidism, neuroinflammation, psychosis, thyroid peroxidase antibodies

## Abstract

Hypothyroidism, particularly in the context of autoimmune conditions such as Hashimoto’s thyroiditis, can lead to a variety of neuropsychiatric symptoms. While cognitive and mood disturbances are frequently encountered, psychosis is a rare and often unexpected complication. This case report describes a middle-aged female with a history of thyroid dysfunction who presented with acute psychosis, including delusions and suicidal ideation. Laboratory investigations confirmed a diagnosis of Hashimoto’s thyroiditis. The patient, already on thyroid hormone replacement, was treated with olanzapine, an antipsychotic, leading to a marked improvement in her psychiatric condition. This case underscores the importance of considering underlying thyroid dysfunction in patients presenting with complex psychiatric symptoms and highlights the role of early intervention in preventing prolonged psychiatric complications.

## Introduction

Hypothyroidism, especially when caused by Hashimoto’s thyroiditis, is a condition characterized by chronic inflammation and destruction of the thyroid gland due to autoimmune processes. It is the most common cause of hypothyroidism in developed countries. The condition primarily presents with fatigue, weight gain, and cold intolerance but can also lead to neuropsychiatric manifestations, including cognitive impairment, depression, and in rare cases, psychosis [1]. Psychosis in the context of hypothyroidism is referred to as "myxedema madness" and is a severe but rare complication of thyroid hormone deficiency. Psychosis occurs in approximately only 5% of cases involving Hashimoto’s thyroiditis [2]. Patients may experience delusions, hallucinations, and disorganized thinking, which can be easily mistaken for primary psychiatric disorders, making timely diagnosis challenging [3].

The link between hypothyroidism and psychosis remains poorly understood, but research suggests that thyroid hormones play a crucial role in brain function and neurotransmitter regulation. Disruptions in these hormones may lead to imbalances in neurotransmitter systems such as serotonin, dopamine, and norepinephrine, which are key to mood regulation and cognitive function. Furthermore, in Hashimoto’s thyroiditis, autoimmune mechanisms may contribute to neuroinflammation, further exacerbating psychiatric symptoms. Elevated levels of thyroid peroxidase antibodies (anti-TPO), commonly seen in Hashimoto's thyroiditis, suggest that immune-mediated processes may possibly affect the central nervous system and the balance of neurotransmitters, causing psychiatric disturbances [4].

This case report examines the development of first-onset psychosis in a 48-year-old female patient with hypothyroidism due to Hashimoto’s thyroiditis, despite being on thyroid replacement therapy (levothyroxine). The case highlights the importance of considering thyroid dysfunction as a differential diagnosis in patients presenting with new-onset psychosis, particularly outside the usual peak age of onset, when psychiatric symptoms emerge without any past psychiatric history or family history of mental illness. Recognizing the connection between autoimmune thyroiditis and psychiatric symptoms is crucial for guiding appropriate treatment and achieving symptom resolution in such rare cases [5].

## Case presentation

A 48-year-old South Asian female, married with three children, with no previous psychiatric history, was admitted to a tertiary psychiatric hospital following a two-week history of worsening psychiatric symptoms. She was on regular levothyroxine 125 mcg daily for her hypothyroidism. These psychiatric symptoms included persecutory delusions, suicidal ideation, and erratic behavior, which had escalated over the preceding three days, with increasing suspicions towards her family, disrupted sleep, and diminished appetite.

Her family reported that her behavior had been gradually deteriorating, becoming more turbulent after a recent trip to her home country. The patient began expressing intense fears that her in-laws were using black magic to harm her. She developed fixed delusions centered around family persecution and was convinced her in-laws, who were responsible for the murders of two family members, falsely implicated her in these crimes. Her mood became severely anxious and she was in constant distress, and she exhibited social withdrawal and an inability to care for herself. Three days prior to admission, after locking herself in the bathroom, she attempted to drown herself in the bathtub, later revealing plans to commit suicide at a nearby beach after the failure of the first attempt by drowning. Alarmed by her worsening condition, her family contacted emergency services, and she was immediately taken to a general medical facility where she was medically cleared and then transferred to our psychiatric facility for psychiatric evaluation and management.

Upon admission, the patient appeared sedated due to medication administered at the referring hospital. Her initial physical examination was unremarkable, with stable vital signs, including a temperature of 36.7°C, heart rate of 76 bpm, and blood pressure of 125/78 mmHg. A mental status examination was deferred initially due to sedation, and she was placed on close observation for risk to herself. Given her known history of hypothyroidism and ongoing psychiatric symptoms, thyroid function tests (TFTs) and anti-TPO antibody testing were ordered alongside other routine laboratory investigations (Table 1). Almost all lab investigations were insignificant except for TPO antibodies.

**Table 1:** Laboratory investigations conducted on admission. x10³/mcL: thousand cells per microliter; x10⁶/µL: million cells per microliter; g/dL: grams per deciliter; %: percentage; fL: femtoliters; pg: picograms; g/dL: grams per deciliter; x10³/mcL: thousand cells per microliter; mmol/L: millimoles per liter; µmol/L: micromoles per liter; IU/L: international units per liter; U/L: units per liter; pmol/L: picomoles per liter; µIU/mL: micro-international units per milliliter; mIU/L: milli-international units per liter; mm/hr: millimeters per hour; mg/L: milligrams per liter; eGFR: estimated glomerular filtration rate; ALT: alanine aminotransferase; AST: aspartate aminotransferase; hCG: human chorionic gonadotropin; IgM: immunoglobulin M; MDMA: 3,4-methyl​enedioxy​methamphetamine, RPR: Rapid Plasma Reagin; N/A: not applicable.

Group	Investigation	Value with Units	Flags	Normal Range
Anemia profile	Ferritin level	8.40 ng/mL	LOW	10.00-291.00
	Iron level	10.70 umol/L	-	9.00-30.40
Complete blood count	White blood cell count	6.08 x10(3)/mcL	-	4.00-10.00
	Red blood cell count	4.15 x10(6)/mcL	-	3.80-4.80
	Hemoglobin	10.90 gm/dL	LOW	12.00-15.00
	Hematocrit	34.90%	LOW	36.00-46.00
	Platelet	209.00x10(3)/mcL	-	150.00-450.00
Other hematology	Erythrocyte sedimentation rate (ESR)	22.00 mm/hr	HIGH	0.00-20.00
Electrolytes and renal profile	Hemoglobin A1c	5.30%	-	4.80-6.00
	Sodium	139 mmol/L	-	136-145
	Potassium	3.67 mmol/L	-	3.50-5.10
	Chloride	102 mmol/L	-	98-107
	Carbon dioxide (CO2)	28.1 mmol/L	-	21.0-32.0
	Anion gap	9 mmol/L	-	5-15
	Creatinine	91 umol/L	-	53-97
	Uric acid	277 umol/L	-	155-357
	Random glucose	4.5 mmol/L	-	3.9-11.1
	eGFR	67	-	
Liver profile	Total protein	72 gm/L	-	64-82
	Albumin level	34.9 gm/L	LOW	38.0-56.0
	Total bilirubin	6.4 umol/L	-	3.0-17.0
	ALT	18 IU/L	-	14-59
	AST	9 U/L	LOW	15-37
	Alkaline phosphatase	57.00 IU/L	-	46.00-116.00
	Gamma glutamyl transferase	19 IU/L	-	5-55
	Amylase level	32 IU/L	-	25-115
	Cholesterol	5.47 mmol/L	-	
	Triglycerides	0.79 mmol/L	-	
	High-density lipoprotein cholesterol (HDL)	1.67 mmol/L	-	
	Low-density lipoprotein cholesterol (LDL)	3.11 mmol/L	-	
Cardiac profile	Creatine kinase	142 IU/L	-	26-192
Miscellaneous chemistry	Lipase	31.00 IU/L	-	16.00-77.00
	25-hydroxy vitamin D	44.85 nmol/L	-	
Hormones	Prolactin	2,152 uIU/mL	-	
Inflammatory markers	C-reactive protein (CRP)	3.1 mg/L	HIGH	0.0-3.0
Tumur markers	Beta hCG	2.00 IU/L	-	
HIV screening	HIV antibodies (1&2) and p24 antigen	Non-reactive	-	
	Hep B core IgM	Non-reactive	-	
	Hep Be antibody	Non-reactive	-	
	Hep Bs antibody	Non-reactive	-	
	Hep C antibody	Non-reactive	-	
Syphilis	RPR	Non-reactive	-	
Urine toxicology	Amphetamine	Negative	-	
	Benzodiazepine	Negative	-	
	Cocaine	Negative	-	
	Opiate	Negative	-	
	Barbiturate	Negative	-	
	Heroin	Negative	-	
	MDMA	Negative	-	
	Methadone	Negative	-	
	Phencyclidine	Negative	-	
	Tramadol	Negative	-	
	Buprenorphine	Negative	-	
	Cannabis	Negative	-	

By the second day of hospitalization, the patient was alert enough for a detailed mental status examination. She displayed significant persecutory delusions focused on her in-laws and continued to express paranoia about being harmed. She was fully conscious, with stable levels of consciousness throughout her hospital stay. She was alert and fully oriented to time, place, and person. Her attention appeared grossly intact. Despite coherent speech and intact memory, her insight and judgment were impaired. She was started on olanzapine 10 mg once daily to manage her psychosis.

On the third day, her thyroid function tests revealed a normal thyroid stimulating hormone (TSH) of 0.58 mIU/L, with normal free T3 (4.31 pmol/L) and free T4 (19.77 pmol/mL) levels. However, her anti-TPO antibody levels were markedly elevated at 415.20 U/mL, indicating Hashimoto’s thyroiditis (Table 2). This suggested an autoimmune process contributing to her psychiatric symptoms despite her being on levothyroxine. Consequently, her levothyroxine dose was maintained at 125 mcg per day, and the focus shifted to managing her psychiatric condition.

**Table 2:** Results of thyroid function tests.

Investigation	Value w/Units	Flags	Normal range
Free T4	16.09 pmol/L		9.00-20.00
Free T3	4.31 pmol/L		3.50-6.50
Thyroid stimulating hormone (TSH)	0.58 mIU/L		0.55-4.78
Thyroid peroxidase (TPO) antibody	415.20 U/mL	HIGH	0.00-60.00
Thyroglobulin antibody	0.90 U/mL		0.00-1.30

By the fourth day, the patient showed mild improvement in mood and a reduction in the intensity of her delusions. She remained cooperative, with improved sleep and appetite. Her suicidal ideation had diminished, though her insight into her condition was still limited.

By the fifth day, her delusions had fully resolved. She no longer exhibited paranoid thoughts and acknowledged the irrationality of her previous beliefs. Her mental state stabilized, and she was able to participate in discussions about her treatment and discharge plans. A family meeting was conducted, where the patient expressed regret for her earlier actions and demonstrated a better understanding of her health condition.

The patient was discharged after eight days of hospitalization with a diagnosis of Psychotic Disorder Due to a General Medical Condition. She was discharged home on levothyroxine 125 mcg daily to continue managing her thyroid function and olanzapine 10 mg at bedtime for psychiatric stability. Psychoeducation was provided to the patient and her family regarding the importance of medication adherence for both her thyroid condition and mental health. She was scheduled for outpatient follow-up appointments with both endocrinology and psychiatry within one week of discharge to monitor her thyroid levels and mental status. Her family was also advised to watch for early signs of relapse and ensure she adhered to her treatment regimen.

## Discussion

The link between hypothyroidism and psychosis is well-established, particularly in cases of severe hypothyroidism. Psychosis in hypothyroid patients is often characterized by paranoid delusions, hallucinations, and cognitive deficits. However, this case is unique because the patient presented with psychosis despite having normal thyroid hormone levels (TSH, T3, and T4) and was on levothyroxine 125 mcg daily. This raises the question of whether subclinical hypothyroidism or an autoimmune process related to Hashimoto’s thyroiditis played a role in her psychiatric presentation [2,5].

Hashimoto’s thyroiditis, an autoimmune condition, is characterized by chronic inflammation of the thyroid gland, often leading to hypothyroidism. While patients with severe hypothyroidism (often referred to as myxedema madness) present with clear psychiatric symptoms, including psychosis, the presentation of psychiatric symptoms in cases where thyroid function tests are normal remains rare and clinically significant. Studies have shown that patients with normal thyroid hormone levels but elevated anti-TPO antibodies can be at risk of developing neuropsychiatric symptoms such as psychosis [1,5]. In this patient, her elevated anti-TPO antibodies indicated ongoing autoimmune activity, suggesting that her psychosis could be linked to subtle neuroinflammation rather than severe thyroid hormone dysfunction [3,6].

Psychiatric symptoms, including psychosis, often emerge as thyroid dysfunction worsens. However, in this case, where thyroid hormone levels remained normal except for raised anti-TPO antibodies, undetected or underappreciated mild hypothyroidism or subclinical hypothyroidism may be a contributing factor. Subclinical hypothyroidism, characterized by elevated TSH with normal T3 and T4 levels, can present with vague symptoms such as fatigue, mood changes, or cognitive difficulties, which are easily overlooked when psychosis dominates the clinical picture [2,3].

The autoimmune nature of Hashimoto’s thyroiditis is an important factor in understanding the psychosis in this case. Elevated anti-TPO antibodies, even with normal thyroid function, may provoke neuroinflammatory processes that affect serotonin and dopamine pathways, critical to mood and cognition [3,7]. This suggests that the autoimmune activity was probably a significant contributor to the patient's psychosis, even in the absence of overt hypothyroid symptoms or neurological manifestations [4,7].

Several case studies have documented similar occurrences of psychosis in patients with Hashimoto’s thyroiditis who were on thyroid hormone replacement therapy. One study noted a case of myxedema psychosis, where psychiatric symptoms persisted despite normal thyroid hormone levels, the psychosis resolved after adjusting the levothyroxine dose and using antipsychotic medications [1,4]. These findings align with our patient’s presentation, where her psychiatric symptoms improved significantly within a week of treatment with olanzapine 10 mg daily, further indicating that her psychosis was driven by organic factors related to her autoimmune thyroid condition rather than a primary psychiatric disorder, which may have taken much longer to resolve [6,8].

One of the key distinguishing factors in this case is the late onset of psychosis at age 48, which is uncommon for primary psychiatric conditions such as brief psychotic disorder, schizophrenia, or bipolar disorder, all of which typically present earlier in life. In women, the mean age of onset of schizophrenia is 25-35 years old [9], whereas for bipolar disorder, it is around 21 years of age [10,11].

The absence of a prior psychiatric history, family history of mental illness, or substance use further supports the notion that the psychosis was secondary to an underlying medical condition rather than a primary psychiatric illness [8,12]. Psychosis linked to hypothyroidism or Hashimoto’s thyroiditis often responds well to thyroid hormone replacement and antipsychotic treatment, as seen in this case [13].

Finally, the patient’s rapid recovery within eight days of starting antipsychotic medication, along with ongoing thyroid hormone replacement therapy, highlights the importance of timely and appropriate management. Though her thyroid hormone levels were normal, it may be important to continue monitoring her thyroid function and adjust the levothyroxine dosage if necessary to prevent future psychiatric relapses. The interplay between thyroid autoimmunity and psychiatric symptoms suggests that more careful management of both psychiatric and endocrine aspects is essential in such cases [4,13].

## Conclusions

This case underscores the rare but significant association between Hashimoto’s thyroiditis, normal thyroid function, and psychosis. The patient’s elevated anti-TPO antibodies, despite normal TSH, T3, and T4 levels, suggest that autoimmune activity likely triggered neuroinflammation, leading to psychosis. The absence of neurological symptoms, combined with her late-onset psychosis and rapid response to antipsychotic treatment, strongly supports the idea that this was not a primary psychiatric disorder. This case highlights the importance of considering autoimmune thyroiditis as a potential cause of psychosis in patients with underlying thyroid disease despite normal thyroid function, especially when psychiatric symptoms emerge unexpectedly. Further research is needed to better understand the mechanisms linking thyroid dysfunction and psychiatric disorders, as well as to refine treatment strategies for patients with hypothyroidism-induced psychosis.
